# Phylogenetic and biological features of severe fever with thrombocytopenia syndrome virus from multi-species infections in eastern China

**DOI:** 10.1128/spectrum.03573-25

**Published:** 2026-07-13

**Authors:** Hao Wang, Dali Xu, Chengyunxiao Li, Xueying Tian, Bo Pang, Ti Liu, Chunhong Yin, Zengqiang Kou

**Affiliations:** 1 School of Public Health, Shandong Second Medical University 372527 , Weifang, Shandong, China; 2 Infectious Disease Prevention and Control Institute, Shandong Center for Disease Control and Prevention 373247, Jinan, Shandong, China; 3 Shandong Provincial Key Laboratory of Intelligent Monitoring, Early Warning, Prevention and Control for Infectious Diseases, Jinan, Shandong, China; 4 School of Public Health, Shandong First Medical University 518873, Jinan, Shandong, China; National Chung Hsing University, Taichung, Taiwan

**Keywords:** SFTS virus, phylogenetics, multi-species transmission, reassortment and recombination, viral replication

## Abstract

Severe fever with thrombocytopenia syndrome (SFTS), caused by SFTS virus (SFTSV), is an emerging tick-borne disease with high mortality and complex transmission dynamics. Since its first identification in China, SFTS has become an endemic infectious disease with continuously expanding geographic distribution and increasing incidence, posing a growing public health concern. In this study, we performed multi-species surveillance, virus isolation, whole-genome sequencing, and phenotypic characterization of 24 SFTSV strains obtained from humans, ticks, and fur-bearing animals in Shandong Province between 2024 and 2025. Phylogenetic analysis revealed co-circulation of four genotypes, with genotype D predominating, and identified eight reassortment and eight recombination events, indicating active genomic reshuffling. Comparative genomic analysis demonstrated high similarity between human- and fur animal-derived SFTSV, suggesting potential cross-species transmission. In *vitro* experiments further confirmed genotype-dependent replication efficiency, with genotype D exhibiting significantly superior viral proliferation. Collectively, these findings highlight the complex genetic diversity, evolutionary dynamics, and multi-species transmission potential of SFTSV, emphasizing the need for integrated genomic and phenotypic surveillance to inform public health interventions.

**IMPORTANCE:**

Severe fever with thrombocytopenia syndrome virus (SFTSV) continues to cause sporadic and clustered infections in East Asia, but its mechanisms of interspecies transmission and local evolution remain poorly defined. This study represents one of the most comprehensive multi-host investigations of SFTSV in eastern China, integrating laboratory diagnosis, genomic surveillance, and biological characterization. By isolating and comparing viral strains from humans, ticks, and fur-bearing animals, we demonstrate genotype-dependent replication efficiency and provide molecular evidence for direct animal-to-human transmission. These findings expand the understanding of SFTSV ecology and evolution, underscore the diagnostic value of multi-source sample testing, and highlight the need for integrated “One Health” surveillance strategies to prevent zoonotic spillover and human infection.

## INTRODUCTION

Severe Fever with Thrombocytopenia Syndrome (SFTS) is an emerging zoonotic infectious disease first identified in rural areas of Hubei and Henan provinces in China in 2009 (1). The causative agent, Severe Fever with Thrombocytopenia Syndrome Virus (SFTSV), also referred to Dabie Bandavirus (DBV), belongs to the genus *Bandavirus* within the family *Phenuiviridae* (2). Clinically, infection is characterized by acute fever, thrombocytopenia, and leukopenia, while severe cases may progress to bleeding tendencies, multi-organ failure, and high fatality rates (3). Multiple transmission routes have been identified, including tick bites, direct contact with blood or body fluids, and mucosal exposure (4–6). SFTSV exhibits remarkable host versatility, infecting humans, domestic livestock (goats, cattle, pigs), companion animals (dogs, cats), wildlife (deer, hedgehogs, rodents), and, more recently, fur-bearing animals such as mink and fox (5–15).

Morphologically, SFTSV particles are spherical (80–110 nm in diameter), enveloped, and exhibited peripheral filaments (16). The SFTSV genome consists of three single-stranded negative-sense RNA segments, designated L, M, and S (16). The L segment encodes RNA-dependent RNA polymerase (RdRp) required for viral transcription and replication (17). The M segment is translated into a glycoprotein precursor (GP) that is post-translationally cleaved into the Gn and G for host cell entry and virion assembly (18). The S segment employs an ambisense strategy to produce the nucleoprotein (NP) and non-structural protein (NSs) (19). Research on the molecular evolution and genetic characteristics of SFTSV remains limited, and no consensus has been reached regarding its genotype classification. While some studies have clustered SFTSV into three lineages (20), others have proposed five (21, 22), six (23), or even two clades comprising eight genotypes (24). Similar to other trisegmented RNA viruses, SFTSV demonstrates elevated nucleotide substitution rates and is prone to frequent recombination and segment reassortment (25). These evolutionary processes, compounded by variations in sampling strategies and analytical methodologies, underscore the extensive genetic diversity of SFTSV and the widespread co-circulation of phylogenetically distinct viral lineages.

Since its initial detection in China in 2010, SFTS has been reported in multiple provinces with several cases and fatalities. Shandong Province, located in eastern China, constitutes a high-incidence area for SFTS, with cumulative case numbers recorded over several years (26). Although the disease is considered endemic, the molecular epidemiology and evolutionary dynamics of SFTSV within Shandong Province remain incompletely elucidated (27). The role of fur-bearing animals in sustaining viral circulation and facilitating cross-species transmission remains unclear (28). Filling these knowledge gaps is crucial for elucidating host-vector-virus interactions and providing scientific evidence for local prevention and control measures.

In this study, we aimed to characterize the genotypic distribution and evolutionary dynamics of viral strains in Shandong Province, to investigate the potential role of fur-bearing animals in the transmission of SFTSV to humans, and to assess the biological properties of recently circulating isolates. To this end, we analyzed serum samples from SFTS patients, tick specimens, and mink samples collected between April 2024 and June 2025, combined with viral isolation, molecular evolutionary analyses, and cell-based infection assays. By integrating epidemiological, genetic, and biological data, this work provides new insights into the genetic diversity, evolutionary trends, and cross-species infection potential of SFTSV in Shandong, thereby offering a more comprehensive understanding of its molecular epidemiology and informing future surveillance and control strategies.

## MATERIALS AND METHODS

### Sample collection and processing

Serum samples were obtained from 19 laboratory-confirmed SFTS cases in Shandong Province, 2024–2025. Serum or tissue samples (heart, liver, spleen, lung, kidney) from 60 clinically suspected minks were collected from three fur animal farms in Shandong. Additionally, 2,473 ticks were collected from national surveillance sites in Shandong Province (2024), and one tick was removed from a dog associated with one infected case. Ticks were pooled by region and physiological state (adult, blood-fed, partially blood-fed, weak), yielding 132 pools. For mink, the five organs from each animal were combined into a single homogenate. All serum and tissue samples were stored at −80°C. Tick and mink samples were morphologically identified, homogenized on dry ice, suspended in DMEM with 2% FBS and 1% penicillin–streptomycin (S/P), and centrifuged at 8,000 × *g* for 5 min at 4°C. Supernatants were collected for downstream analyses.

### Nucleic acid extraction and SFTSV detection

Total RNA was extracted from serum, tick, and tissue homogenates using commercial viral nucleic acid extraction kits (BioPerfectus, China) according to the manufacturer’s instructions. SFTSV infection was confirmed by Real-time RT-PCR was performed using a fluorescent probe-based kit (Dana Gene, China). Ct ≤35 is deemed positive for SFTSV.

### Whole genome sequencing

Viral RNA was extracted from clinical samples using the QIAamp Viral RNA Kit (Qiagen, 52906). Whole-genome enrichment of SFTSV was performed with a multiplex PCR-based capture kit specific for SFTSV (Baiyi Tech Co., Ltd., BK-WSFTSV2024). The resulting amplicons were purified with AMPure XP beads (Beckman Coulter, A63880) and quantified using Qubit dsDNA HS Assay Kit (Thermo Fisher Scientific, Q32854). Libraries were constructed through enzymatic fragmentation, adapter ligation, and index PCR, followed by purification, quantification, pooling, and denaturation. Sequencing was performed on the Illumina NextSeq 2000 platform with the MiSeq V2 Reagent Kit (Illumina, MS120 102-2001) to generate paired-end reads. Consensus genomes were assembled using a reference-based method in QIAGEN CLC Genomics Workbench (version 23.0.3).

### Phylogenetic analysis

A set of representative full-length SFTSV genome sequences covering all six known SFTSV genotypes (A–F) was randomly derived from the NCBI GenBank database. These sequences, together with the 24 genomes obtained in this study, were used for phylogenetic tree construction. Multiple sequence alignments were performed using MAFFT version 7 (https://mafft.cbrc.jp/alignment/server/index.html). Poorly conserved regions were identified and trimmed using the trimAL Wrapper module implemented in TBtools v2.225.

### Homology and analysis of amino acid mutation sites

Nucleotide and amino acid sequence alignment were analyzed using BioAider 1.727 software. The amino acid substitution sites within the four viral proteins (RdRp, GP, NP, and NSs) were analyzed with MEGAX software, comparing with the reference sequence HN6 strain (29) (GenBank Accession No. for the L, M, and S fragments: HQ141595, HQ141596, and HQ141597, respectively). Using SPSS software (version 27.0), the Mann-Whitney *U* test was employed to compare the mean amino acid mutation counts between 2025 and 2024, thereby assessing the disparity between the two years.

### Recombination analysis

Potential recombination events were assessed using RDP4 software (version 4.100) with seven detection methods, including RDP, MaxChi, BootScan, 3Seq, GENECONV, Chimaera, and SiScan. Recombination events were preliminarily identified when at least four methods yielded *P* ≤ 0.05.

### Virus isolation and cultivation

SFTSV RNA positive samples, including serum, tick, and animal tissue homogenates, were diluted 1:10 in DMEM supplemented with 2% FBS and 1% P/S and filtered through a 0.22 μm membrane. The filtrates were inoculated onto Vero-E6 cell monolayers and incubated at 37°C, 5% CO₂. Cultures were monitored daily for cytopathic effects (CPEs). In the absence of visible CPEs, supernatants were harvested at 6 days post infection and subjected to three sequential blind passages. Viral production was confirmed by RT-PCR, and virus-containing supernatants were collected, aliquoted, and stored at −80°C for subsequent analyses.

### Immunofluorescence assay

Vero-E6 cells were infected with third-passage SFTSV stock at an MOI of 0.01. After 3 days of infection, cells were fixed with 4% paraformaldehyde (PFA) for 30 min, permeabilized with 0.2% Triton X-100 for 15 min, and blocked with 5% Bovine Serum Albumin (BSA) diluted in PBS. Cells were then incubated with anti-NP polyclonal antibody for 2 h, followed by Alexa Fluor 594-conjugated goat anti-mouse antibody for 1 h in the dark. Nuclei were counterstained with DAPI (1:1,000) for 10 min. Fluorescence signals were imaged under a microscope (Leica DMi8) using identical exposure settings.

### Transmission electron microscopy

Supernatants from cultured virus fraction were centrifuged at 100,000 × *g* for 1 h to concentrate viral particles. Pellets were gently resuspended in PBS, and 1–2 drops of suspension were applied to copper grids for 1 min. Excess liquid was removed with filter paper, followed by negative staining with 2% phosphotungstic acid for 1 min. Grids were blotted dry and examined by transmission electron microscopy to visualize viral morphology.

### Viral titration by immunoblot assay

Vero-E6 cells were seeded into a 24-well plate (1.5 × 10⁵ cells per well) and cultured overnight until approximately 80% confluence. SFTSV virus stock solutions were serially diluted 10-fold (10⁻¹ to 10⁻⁵) in duplicate. Following the removal of culture medium, diluted virus suspensions were added to cells and incubated at 37°C in 5% CO₂ for 1 h, with gentle agitation every 15 min. After adsorption, the inoculum was removed, and 500 μL of pre-warmed carboxymethylcellulose (CMC) semi-solid medium was added to each well. Cultures were maintained for 4 days, and then the CMC overlay was discarded. Cells were fixed with methanol: acetone (1:1) for 15 min, washed with PBS, and sequentially incubated with anti-SFTSV NP polyclonal antibody and HRP-labeled secondary antibody. Color was developed using DAB substrate in the dark, and viral foci were counted to determine titers expressed as focus-forming units (FFU).

### Replication dynamics of SFTSV in Vero-E6 cells

Vero-E6 cells were seeded into 24-well plates 1 day prior to infection and infected with three SFTSV isolates (representatives of D/D/D, E/E/E, D/D/E genotypes) at an MOI (multiplicity of infection) of 0.01. After 1 h adsorption at 37°C with 5% CO₂, cells were washed and maintained in 500 μL fresh medium. Supernatants were collected at 0, 8, 24, 48, 72, 96, 120, and 144 h post-infection (two replicates per time point) and stored at −80°C. Viral RNA was extracted using the QIAamp Viral RNA Kit for RNA and quantified by qRT-PCR with the HiScript II One Step qRT-PCR Probe Kit (Vazyme) targeting the SFTSV S segment. The specific primers were as follows: SFTSV-S-Forward (5′- TGTCAGAGTGGTCCAGGATT-3′), SFTSV-S-Reverse (5′- ACCTGTCTCCTTCAGCTTCT-3′), and SFTSV-S-Probe (5′-FAM-TGGAGTTTGGTGAGCAGCAGC-BHQ1-3′). Threshold cycle (Ct) values were analyzed using QuantStudio Design and Analysis Software. Viral titers were calculated based on a standard curve generated from 10-fold serial dilutions of reference RNA extracted from viral stocks of known titers. A two-factor nonparametric analysis of variance was conducted on the FFU values of different SFTSV genotypes at various infection times in Vero-E6 cells.

### Statistical analysis

Viral titers were analyzed using two-way non-parametric analysis of variance (ANOVA) in SPSS (version 27). Pairwise differences in viral titers among the three genotypes at each time point were assessed using the Kruskal-Wallis multiple comparison test in SPSS (version 27). Inter-year differences in the number of amino acid mutations across four SFTSV proteins (RdRp, GP, NSs, NP) from 2024 and 2025 were evaluated using the Mann-Whitney *U* test. *P* values < 0.05 were considered statistically significant and ns indicates not significant. Significance levels are denoted as follows: *P < 0.05, **P < 0.01, ***P < 0.001.

## RESULTS

### Detection and phylogenetic analysis of STFSV derived from Shandong Province

A total of 19 human cases were confirmed as SFTSV-positive via molecular diagnostics. Tick specimens (*n* = 2,473) collected from surveillance sites and one tick removed from a dog associated with an infection case were morphologically identified as *Haemaphysalis longicornis*, with two pools testing positive for SFTSV (1.52%, 2/132). Among 60 serum or tissue samples (heart, liver, spleen, lung, kidney) from mink suspected of SFTSV infection, SFTSV nucleic acid was detected in 2 tissue samples (6.67%) and 24 serum samples (80%), indicating substantial viral circulation in farmed mink (Supplemental data set).

**Fig 1:**
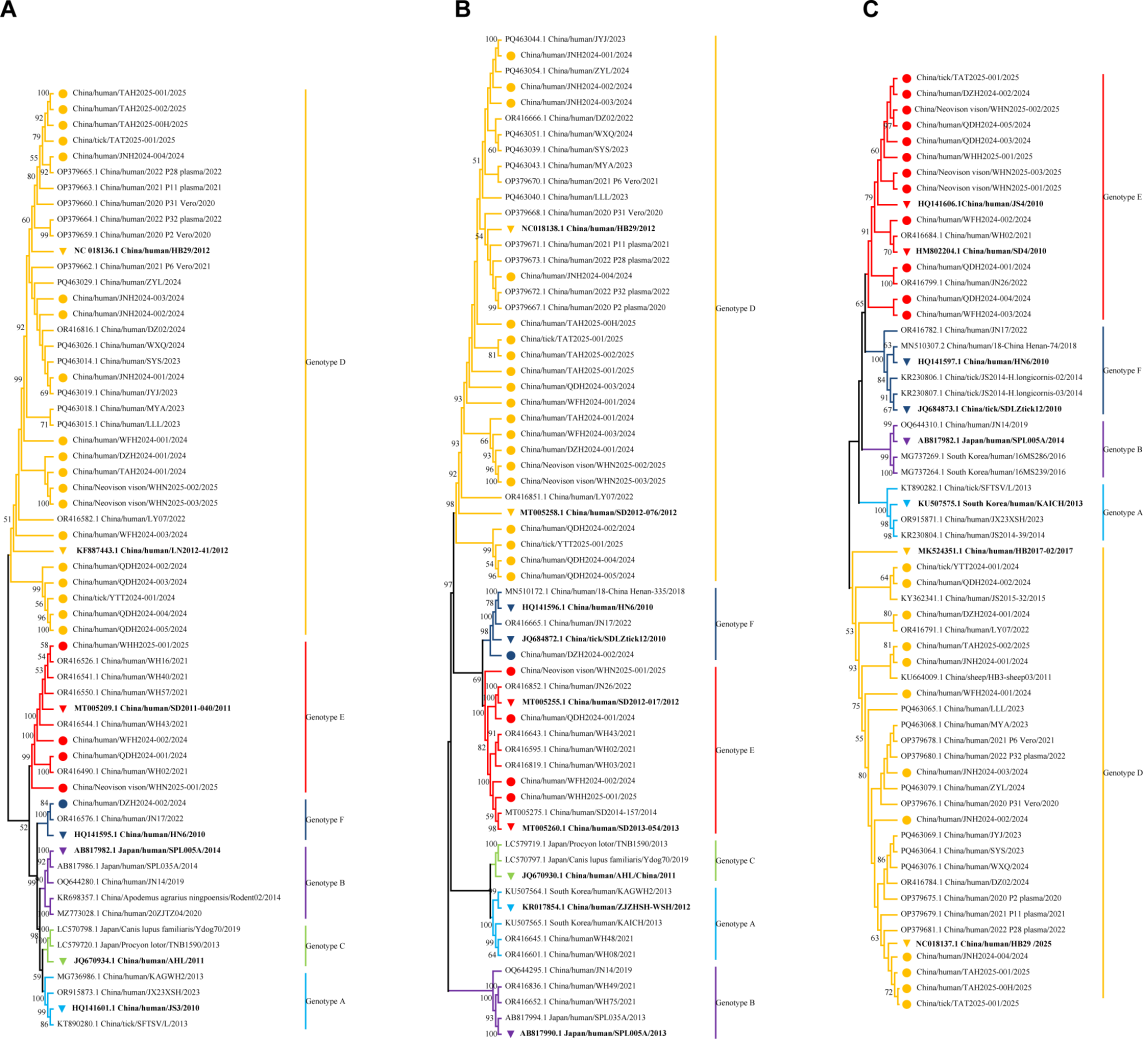
Phylogenetic analysis of SFTSV. Phylogenetic trees were constructed based on the complete nucleotide sequences of the L, M, and S segments from 24 SFTSV isolates, respectively (**A–C**) Red dots indicate genotype E, yellow dots indicate genotype D, and blue dots indicate genotype F. The inverted triangle and bolded notations indicate the reference sequences for each genotype, which were selected based on the analysis in the study by Fu et al. (30).

From all positive samples, 24 representative samples were selected, including 19 SFTS patient sera, 2 tick specimens, and 3 from mink sera (1 from each of the 3 fur animal farms, all with Ct values < 32). Whole-genome sequencing was performed on these 24 representative SFTSV-positive samples, covering human, mink, and tick sequences. Phylogenetic analysis based on the full-length L, M, S segments was performed taking corresponding SFTSV sequences as reference, revealing the co-circulating of multiple genotypes in Shandong Province. Previous research demonstrated that 24 sequences clustered into 3 lineages D, E, and F across the L, M, and S gene phylogenies (14). Specifically, 12 sequences isolated in Jinan (JNH2024-001/002/003/004), Dezhou (DZH2024-001), Weifang (WFH2024-001), Qingdao (QDH2024-002), Taian (TAH2025-001/002/00H), Yantai tick (YTT2024-001), and Taian tick (TAT2025-001) clustered within lineage D across the L, M, and S gene fragments (Fig. 1A through C; Table 1). Four sequences derived from Weifang (WFH2024-002), Qingdao (QDH2024-001), Weihai (WHH2025-001), and mink-derived WHN2025-001 grouped into lineage E (Fig. 1A through C; Table 1).

**TABLE 1:** Basic information for 24 SFTSV isolated sequences

	Sequence name	Collection date	Region	Genome segment	Genotype	Accession no.
1	JNH2024-001	2024-05-22	China: Jinan	L	D	C_AA120303.1
				M	D	C_AA120279.1
				S	D	C_AA120229.1
2	JNH2024-002	2024-09-06	China: Jinan	L	D	C_AA120304.1
				M	D	C_AA120280.1
				S	D	C_AA120230.1
3	JNH2024-003	2024-10-02	China: Jinan	L	D	C_AA120305.1
				M	D	C_AA120281.1
				S	D	C_AA120231.1
4	JNH2024-004	2024-10-23	China: Jinan	L	D	C_AA120306.1
				M	D	C_AA120282.1
				S	D	C_AA120232.1
5	DZH2024-001	2024-06-04	China: Dezhou	L	F	C_AA120301.1
				M	F	C_AA120277.1
				S	E	C_AA120227.1
6	DZH2024-002	2024-06-05	China: Dezhou	L	D	C_AA120302.1
				M	D	C_AA120278.1
				S	E	C_AA120228.1
7	TAH2024-001	2024-07-26	China: Taian	L	D	C_AA120312.1
				M	D	C_AA120288.1
				S	D	C_AA120238.1
8	WFH2024-001	2024-08-12	China: Weifang	L	D	C_AA120317.1
				M	D	C_AA120293.1
				S	D	C_AA120243.1
9	WFH2024-002	2024-07-04	China: Weifang	L	E	C_AA120318.1
				M	E	C_AA120294.1
				S	E	C_AA120244.1
10	WFH2024-003	2024-07-29	China: Weifang	L	D	C_AA120319.1
				M	D	C_AA120295.1
				S	E	C_AA120245.1
11	QDH2024-001	2024-05-09	China: Qingdao	L	E	C_AA120307.1
				M	E	C_AA120283.1
				S	E	C_AA120233.1
12	QDH2024-002	2024-08-03	China: Qingdao	L	D	C_AA120308.1
				M	D	C_AA120284.1
				S	D	C_AA120234.1
13	QDH2024-003	2024-08-31	China: Qingdao	L	D	C_AA120309.1
				M	D	C_AA120285.1
				S	E	C_AA120235.1
14	QDH2024-004	2024-10-09	China: Qingdao	L	D	C_AA120310.1
				M	D	C_AA120286.1
				S	E	C_AA120236.1
15	QDH2024-005	2024-10-22	China: Qingdao	L	D	C_AA120311.1
				M	D	C_AA120287.1
				S	E	C_AA120237.1
16	WHH2025-001	2025-02-17	China: Weihai	L	E	C_AA120320.1
				M	E	C_AA120296.1
				S	E	C_AA120246.1
17	TAH2025-001	2025-06-08	China: Taian	L	D	C_AA120313.1
				M	D	C_AA120289.1
				S	D	C_AA120239.1
18	TAH2025-002	2025-06-08	China: Taian	L	D	C_AA120314.1
				M	D	C_AA120290.1
				S	D	C_AA120240.1
19	TAH2025-00H	2025-05-22	China: Taian	L	D	C_AA120315.1
				M	D	C_AA120291.1
				S	D	C_AA120241.1
20	YTT2024-001	2024-06-15	China: Yantai	L	D	C_AA120324.1
				M	D	C_AA120300.1
				S	D	C_AA120250.1
21	TAT2025-001	2025-06-08	China: Taian	L	D	C_AA120316.1
				M	D	C_AA120292.1
				S	D	C_AA120242.1
22	WHN2025-001	2025-05-28	China: Weihai	L	E	C_AA120321.1
				M	E	C_AA120297.1
				S	E	C_AA120247.1
23	WHN2025-002	2025-05-28	China: Weihai	L	D	C_AA120322.1
				M	D	C_AA120298.1
				S	E	C_AA120248.1
24	WHN2025-003	2025-05-28	China: Weihai	L	D	C_AA120323.1
				M	D	C_AA120299.1
				S	E	C_AA120249.1

**Fig 2:**
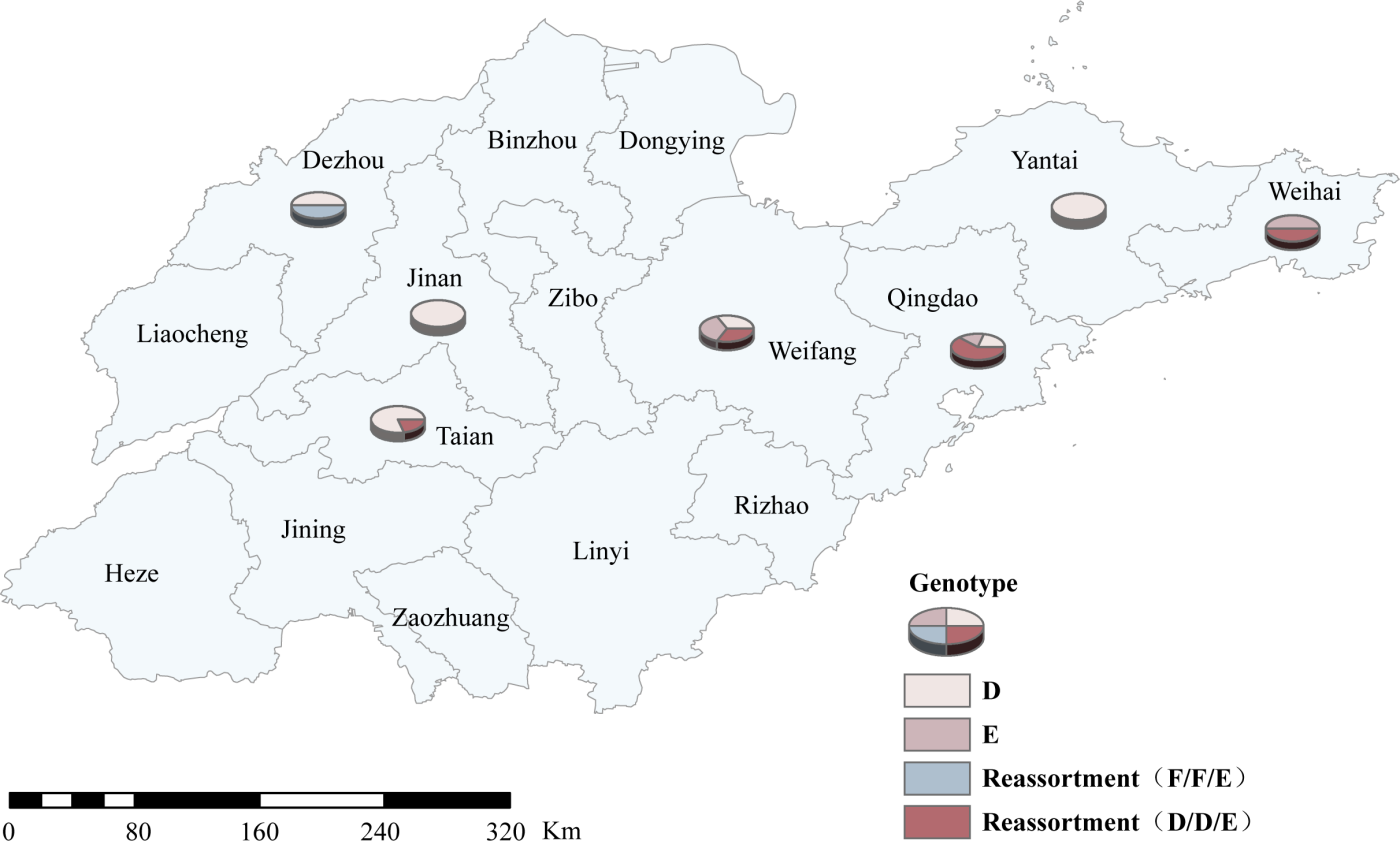
Geographic distribution of SFTS cases and SFTSV genotypes in Shandong Province. The map shows the locations of the 24 samples identified and their corresponding viral genotypes. The base map used in this study was generated using the standard map service provided by the Resource and Environment Science Data Platform, Institute of Geographic Sciences and Natural Resources Research, Chinese Academy of Sciences (https://www.resdc.cn/).

Among the 24 sequences, 8 reassortants were identified, all involving the S segment: Taian (TAH2024-001), Weifang (WFH2024-003), Qingdao (QDH2024-003/004/005), mink-derived Weihai (WHN2025-002/003), and Dezhou (DZH2024-002), with the latter carrying L/M segments from lineage F and the S segment from lineage E. These findings indicate the co-circulation of multiple SFTSV genotypes in eastern China, with lineage D remaining predominant (Fig. 2; Table 1).

### Genomic homology analysis of SFTSV strains

In this study, nucleotide and amino acid sequence homology analyses were conducted on SFTSV strains of different genotypes from Shandong Province. For lineage D sequences, nucleotide (and derived amino acid) similarities with other D-type reference strains were 96.69%–99.95% (99.04%–100%) for the L segment, 94.91%–99.97% (96.28%–100%) for the M segment, and 95.99%–100% (97.11%–100%) for the S segment (Table S2). For lineage E sequences, corresponding similarities were 96.15%–99.89% (99.09%–100%) for the L segment, 92.46%–99.78% (96.55%–99.91%) for the M segment, and 97.05%–100% (95.95%–100%) for the S segment (Table S3). For lineage F sequences, nucleotide (and amino acids) similarities were 95.99%–99.17% (99.38%–99.95%) for the L segment and 97.18%–98.63% (99.35%–99.72%) for the M segment (Table S3).

Of note, one confirmed case of SFTS (WHH2025-001) was reported in Weihai City, in January 2025, suspected to be associated with consumption of fur animals. Whole-genome sequencing of the patient’s serum and a mink serum sample (WHN2025-001) collected from a nearby farm in June 2025 revealed high nucleotide homology of 98.67%, 99.13%, and 100% in the L, M, and S segments, respectively, and amino acid homology of 99.57%, 99.44%, and 100%, respectively (Table S1). These findings suggest a close genetic relationship between human and mink-derived SFTSV strains in this region, implying a potential zoonotic transmission link.

### Amino acid substitution patterns in SFTSV genomes

**Fig 3:**
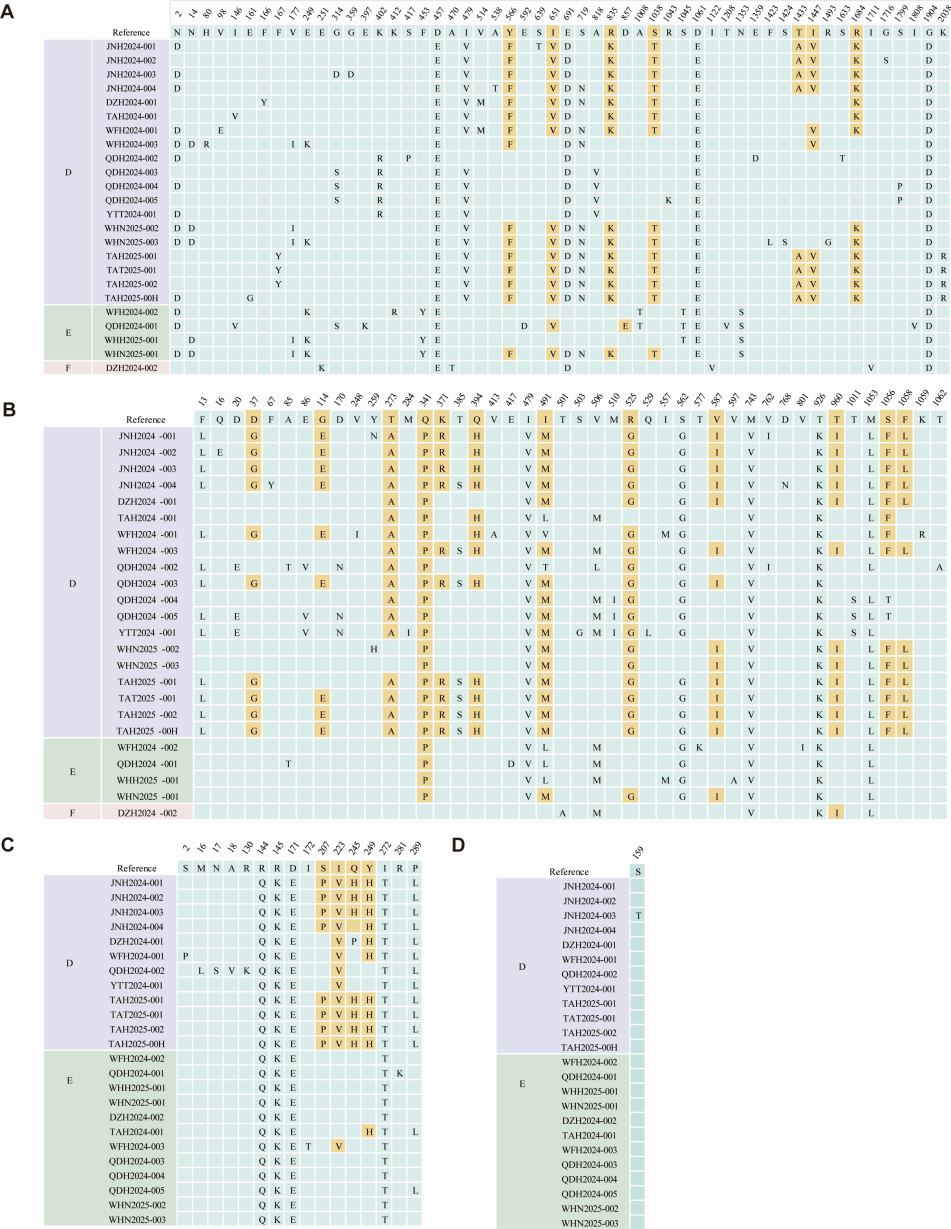
Amino acid mutation analysis of Shandong SFTSV isolates relative to the HN6 reference strain. Amino acid variations were identified using the SFTSV HN6 strain as the reference (GenBank accession numbers: L–HQ141595, M–HQ141596, S–HQ141597). (**A**) RdRp mutations; (**B**) GP mutations; (**C**) NSs mutations; (**D**) NP mutations across 24 Shandong isolates. Yellow-highlighted residues indicate positions associated with fatal SFTS outcomes.

Using the F genotype HN6/China/2010 strain as the reference, amino acid substitution analysis was performed for 4 SFTSV proteins (RdRp, GP, NSs, and NP) across 24 newly obtained full-genome sequences using the MEGAX software. The results revealed a total of 115 amino acid mutations were identified, including 54 (2.59%) in RdRp, 44 (4.10%) in GP, 16 (5.44%) in NSs, and 1 (0.41%) in NP (Fig. 3A through D). RdRp substitutions were primarily clustered between residues 400 and 1,100, with conserved mutations at positions 457 and 1904 detected in all sequences (24/24, 100%) (Fig. 3A). GP substitutions were concentrated between residues 300 and 600, while positions 743, 926, and 1,053 were universally mutated across all sequences (24/24, 100%) (Fig. 3B). NSs amino acid mutations were mainly distributed between 140–170 and 200-250, with positions 144, 145, 171, and 272 consistently mutated in every strain (24/24, 100%) (Fig. 3C). In addition, amino acid mutations among different isolates and genotypes were analyzed in relation to disease fatality. Based on previously reported data, 25 amino acid sites were identified to be associated with case fatality (29). Of these, 24 mutations were detected in the present study (Fig. 3). It is worth noting that a significant proportion of these previously reported loci are found in strains of the D genotype (Fig. 3).

**Fig 4:**
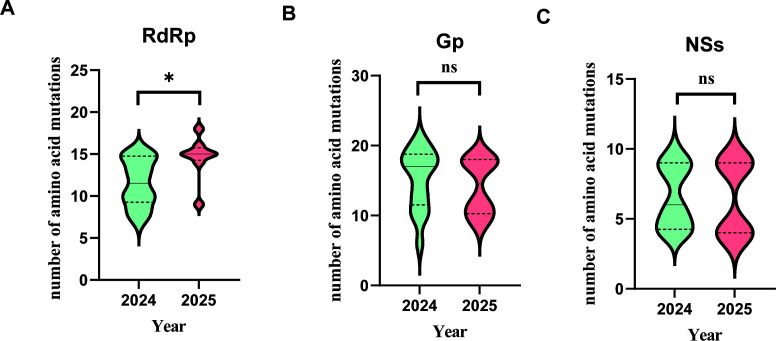
Comparison of amino acid mutations in SFTSV between 2024 and 2025. (**A–C**) Represent RdRp, GP, and NSs, respectively. Mann-Whitney *U* tests were performed in SPSS (v27). RdRp mutations were higher in 2025 than in 2024 (*P* = 0.015, *Z* = −2.43), while GP and NSs showed no significant differences. *P < 0.05, ns, not significant.

Further comparative analysis of the average number of amino acid mutations across the four proteins in Shandong strains isolated in 2024 and 2025 revealed that the mean mutation counts for RdRp, GP, NSs, and NP in 2024 were 12, 15, 7, and 1, respectively, whereas in 2025, these counts were 15, 14, 7, and 0, respectively (Fig. 4A through D). Statistical analysis demonstrated that the number of RdRp substitutions in 2024 was significantly lower than in 2025, indicating an accumulation of amino acid changes in the polymerase gene over time (Fig. 4A).

### Recombination and selection pressure analysis

Using RDP4 (v4.100) to perform recombination analysis on SFTSV sequences from Shandong Province and this study, eight potential recombination events were identified in the newly obtained SFTSV genomes. From the perspective of the L fragment, the strains likely to have undergone recombination include WHN2025-001, WHN2025-002, DZH2024-002, DZH2024-001, and TAH2025-002 (Table 2). In the M fragment, strains that may have undergone recombination include WHN2025-001, WHN2025-002, and DZ2024-002 (Table 2).

**TABLE 2:** Predicted recombinant SFTSV strains in the data set

Strain	RDP	GENECONV	BootScan	MaxChi	Chimera	SiScan	3Seq	SimPlot	Major parent	Minor parent
WHN2025-001-L	−	+	+	+	+	+	+	+	WHH2025-001-L	TAH2025-002-L
WHN2025-002-L	−	+	+	+	+	+	+	+	WHH2025-001-L	TAH2025-00H-L
TAH2025-002-L	+	+	+	+	+	+	+	+	2021_P11-L	WHH2025-001-L
DZH2024-002-L	+	+	−	+	+	+	+	+	JN17-L	WFH2024-001-L
DZH2024-001-L	+	+	+	+	+	+	+	+	ZYL-L	WH16-L
WHN2025-001-M	+	+	+	+	+	+	+	+	WFH2024-002-M	JNH2024-001-M
WHN2025-002-M	+	+	+	+	+	+	+	+	JNH2024-002-M	SD2013-054-M
DZH2024-002-M	−	+	+	+	+	+	+	+	JN17-M	WH02-M

Selective pressure analysis conducted with MEGA X revealed that all four SFTSV proteins were under purifying selection, with d*N*/d*S* ratios below 1 (RdRp: 0.08; GP: 0.16; NP: 0.01; NSs: 0.14). The relatively higher d*N*/d*S* values observed for GP and NSs suggest that these proteins experience weaker purifying selection and may undergo greater adaptive variation, potentially linked to viral entry and host immune interactions.

### Phenotypic characteristics of SFTSV isolates derived from different genotypes

**Fig 5:**
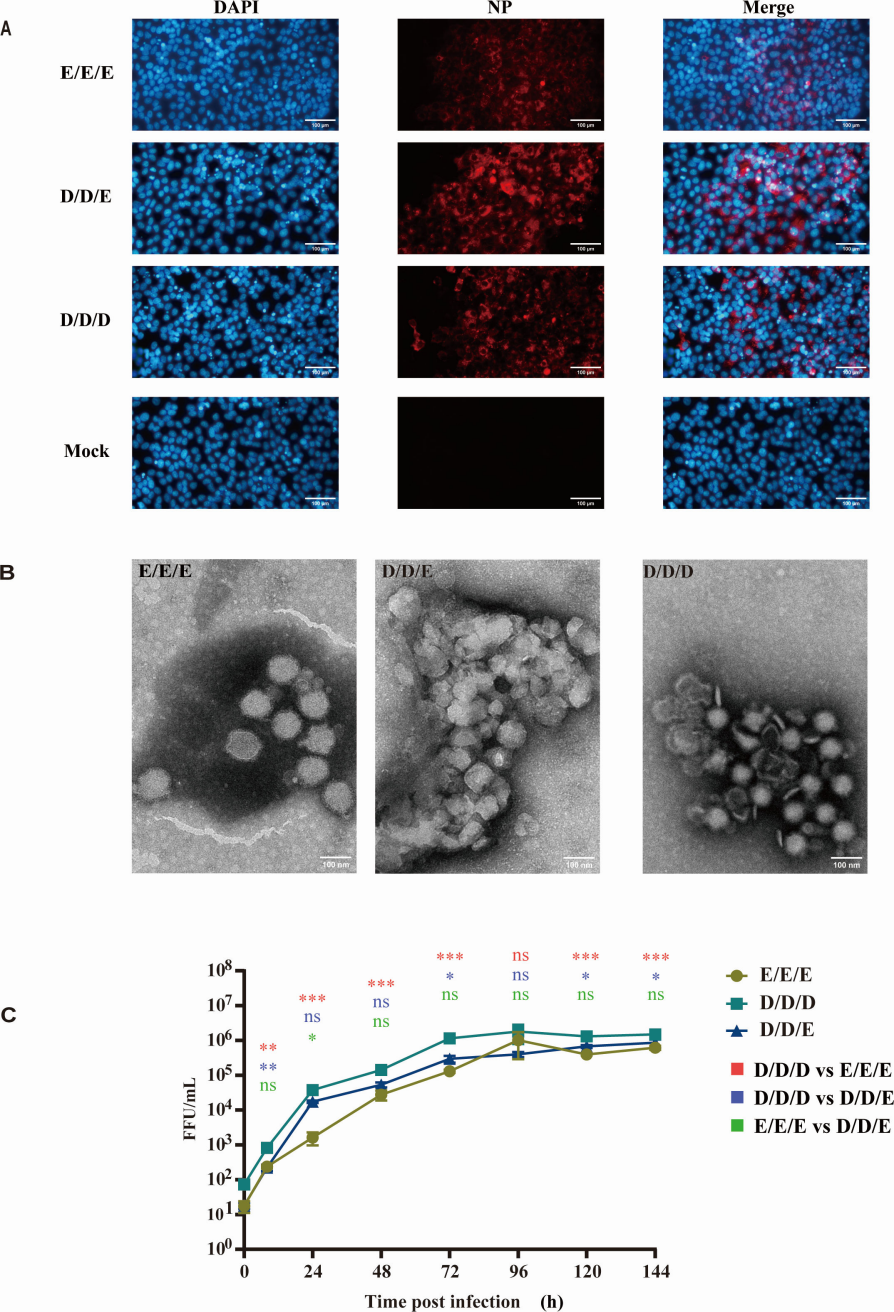
Phenotypic characteristics of SFTSV isolates derived from different genotypes. (**A**) Immunofluorescence assay (IFA) detecting SFTSV NP protein expression in isolated viruses. (**B**) Transmission electron microscopy of purified SFTSV particles. (**C**) Growth curves of SFTSV isolates in Vero-E6 cells. D/D/D, E/E/E, and D/D/E indicate the genotypes of the L, M, and S segments, respectively. Viral supernatants were collected at the indicated time points and titrated. FFU, foci-forming units. Data represent mean ± SEM from three independent experiments, each performed in duplicate. Differences in viral replication among isolates over time were analyzed using two-way nonparametric ANOVA in SPSS (version 27). *P < 0.05, **P < 0.01, ***P < 0.001, ns, not significant.

Three SFTSV strains, representing genotype D (TAH2025-001), genotype E (WFH2024-002), and a reassortment genotype D/D/E (QDH2024-004), were successfully isolated after three to four rounds of blind passaging in Vero-E6 cells. Transmission electron microscopy revealed typical spherical, enveloped virions with surface projections of approximately 80–100 nm in diameter, while immunofluorescence assays confirmed the presence of viral NP antigen in infected Vero-E6 cells, verifying successful viral isolation and replication (Fig. 5A and B).

To investigate phenotypic differences among circulating SFTSV genotypes, three representative isolates, TAH2025-001 (genotype D), WFH2024-002 (genotype E), and QDH2024-004 (reassortant D/D/E), were compared for replication kinetics in Vero-E6 cells. The D-type strain TAH2025-001 exhibited significantly higher replication efficiency than the E-type strain WFH2024-002 across multiple time points (Fig. 5C). The D/D/E reassortant strain QDH2024-004 displayed an intermediate growth pattern between the D- and E-type strains, with viral titers at 12, 72, 120, and 144 h significantly lower than those of TAH2025-001 (Fig. 5C). Comparative amino acid sequence analysis among strains representing D/D/D, E/E/E, and D/D/E genotypes revealed that the D/D/D strain harbored the highest number of substitutions previously associated with severe or fatal SFTS outcomes (Fig. 3; Table 3). Further comparison of the S-segment encoded proteins between D/D/D and D/D/E strains identified five amino acid differences in the NSs protein (P207S, V223I, H245Q, H249Y, and L289P), four of which have been reported to be linked to fatal clinical cases. These findings suggest that variations in the S segment, particularly within the NSs protein, may contribute to genotype-specific differences in viral replication and pathogenic potential.

**TABLE 3:** Amino acid sequence alignment analysis of two representative isolates TAH2025-001 (D/D/D) and WFH2024-002 (E/E/E)

Fragment (Protein)	Amino acid site	Strain name		Mutations related to fatal outcomes
		TAT2025-001 (D/D/D)	WFH2024-002 (E/E/E)	
L (RdRp）	2	N	D	
	167	Y	F	
	249	E	K	
	314	G	G	
	402	K	K	
	412	K	R	
	453	F	Y	
	479	V	I	
	566^*a*^	F	Y	566F^*a*^
	651^*a*^	V	I	651V^*a*^
	691	D	E	
	719	N	S	
	818	A	A	
	835^*a*^	K	R	835K^*a*^
	1008	A	T	
	1038^*a*^	T	S	1038T^*a*^
	1045	S	T	
	1353	N	S	
	1433^*a*^	A	T	1433A^*a*^
	1447^*a*^	V	I	1447V^*a*^
	1684^*a*^	K	R	1684K^*a*^
	1799	S	S	
	2038	R	K	
M (GP）	13	L	F	
	37^*a*^	G	D	37G^*a*^
	273^*a*^	A	T	273A^*a*^
	371^*a*^	R	K	371R^*a*^
	385	S	T	
	394^*a*^	H	Q	394H^*a*^
	491^*a*^	M	L	491M^*a*^
	506^*a*^	V	M	506V^*a*^
	510	M	M	
	525^*a*^	G	R	525G^*a*^
	577	R	K	
	587^*a*^	I	V	587I^*a*^
	801	V	I	
	960^*a*^	I	T	960I^*a*^
	1011	T	T	
	1056^*a*^	F	S	1056F^*a*^
	1058^*a*^	L	F	1058L^*a*^
S (NSs）	207^*a*^	P	S	207P^*a*^
	223^*a*^	V	I	223V^*a*^
	245^*a*^	H	Q	245H^*a*^
	249^*a*^	H	Y	249H^*a*^
	289	L	P	

^*a*^ Amino acid residues reported to be associated with SFTS fatal outcome.

## DISCUSSION

Over the past decade, severe fever with thrombocytopenia syndrome (SFTS) has become increasingly prevalent in China, Japan, and South Korea, posing a serious public health concern (24). In China, SFTS outbreaks have expanded from initially endemic regions of Shandong, Henan, and Jiangsu to a broader geographic range encompassing North, East, and Central China. Both the number of cases and spatial extent of endemic have steadily increased (2). Shandong Province, as one of the earliest and most active endemic regions, represents a key ecological niche where human, tick, and mammalian reservoirs coexist. In this study, we conducted multi-species surveillance, whole-genome sequencing, phylogenetic and phenotypic analyses, and virus isolation of representative SFTSV strains between 2024 and 2025, providing critical insights into the viral genetic diversity, evolutionary dynamics, and cross-species potential transmission patterns, particularly emphasizing the role of fur-bearing animals in zoonotic infection.

Segment-specific phylogenetic analysis using the A–F genotyping system classified the 24 sequenced SFTSV genomes derived from different regions and species into three distinct genotypes D, E, and F, with genotype D being predominant (30). This pattern aligns with previous reports that the C3 genotype is predominant in Shandong (19, 27). The prevalence of genotype D suggests that D-type SFTSV may possess selective advantages in replication efficiency, host adaptability, or immune evasion mechanisms. Understanding the spatial distribution and dominance of specific genotypes is essential for improving regional surveillance, molecular epidemiology, and risk assessment of SFTS outbreaks. The co-circulation of multiple SFTSV genotypes within the same ecological region facilitates opportunities for co-infection of different viral strains in the same host, thereby increasing the likelihood of genetic reassortment, a common evolutionary mechanism among segmented RNA viruses (25, 31, 32). In this study, two types of genetic reassortants (D/D/E and F/F/E) were identified, all involving the reassortment of S segment. Such reassortment events contribute to the genetic diversity and adaptive evolution of SFTSV, potentially influencing its pathogenicity, transmissibility, and epidemic dynamics.

Recombination is considered a potential factor in the evolution of RNA viruses, as it can modify viral antigenicity, virulence, or host adaptability, thereby potentially influencing epidemic dynamics (33). Although the recombination rate in negative-strand segmented RNA viruses such as SFTSV is lower than in positive-strand RNA viruses, accumulating evidence suggests that they may play a significant role in shaping viral evolution through template switching during replication (34–36). In this study, putative recombination signals were identified in the L and M segments using RDP4 (v4.100). These observations may suggest that longer genomic regions could facilitate template switching and genomic rearrangement although this interpretation should be treated with caution (20, 35). Notably, most of the identified recombinant SFTSVs originated from human-derived samples, while several recombination events involved parental contributors from animal-derived sequences. This finding suggests that genetic reassortment and recombination occur not only in human infections but also within animal hosts. The results suggest that further investigation into the roles of different hosts is warranted. These processes may play a significant role in shaping the evolutionary dynamics of SFTSV, driving its genetic diversity, adaptability, and potential emergence of novel lineages with altered pathogenicity or transmission capacity.

Amino acid mutation analysis plays a critical role in elucidating the genetic evolution and pathogenic diversity of SFTSV (37, 38). As the virus adapts to diverse hosts, immune pressures, and ecological conditions, it accumulates non-synonymous mutations that may alter the structural conformation and biological functions of viral proteins. Such alterations can modulate viral replication efficiency, immune evasion capability, and ultimately its pathogenic potential. In the present study, comparative analysis of 24 full-length SFTSV genomes revealed multiple amino acid substitutions distributed across both structural and non-structural proteins. A conserved serine residue was consistently observed at position 962 of the glycoprotein (GP), supporting previous evidence that this site plays a crucial role in membrane fusion and host receptor interaction (39). Moreover, in alignment with earlier reports linking specific amino acid substitutions to disease fatality (29), we identified 24 previously documented mutations associated with severe or fatal outcomes in three fatal cases (JNH2024-003, TAH2025-001, and TAH2025-00H). Isolates collected in 2025 showed more amino acid substitutions in RdRp than those from 2024, suggesting increased sequence variability and ongoing adaptive evolution. These changes may modulate polymerase activity, replication efficiency, or antiviral sensitivity. Together, the findings emphasize the evolutionary plasticity of SFTSV and the need to link genomic variations with clinical and functional outcomes. Analysis of selective pressure across the entire genomic coding regions revealed that d*N*/d*S* ratios were uniformly <1, indicating pervasive purifying selection, consistent with previous observations (22). The average d*N*/d*S* ratio of the GP protein in the M segment is higher than that of the coding regions in the L and S segments, suggesting that it may be subject to relatively weaker selective constraints. However, as these estimates are based on average d*N*/d*S* values at the gene level, they primarily reflect differences in overall conservation between genes and should, therefore, be interpreted with caution. They do not directly prove adaptive evolution or support inferences regarding viral pathogenicity. Further analysis at the codon level and functional studies are required to elucidate the biological significance of these observations.

To further elucidate genotype-specific replication characteristics, we compared the replication kinetics of representative SFTSV isolates of genotypes D/D/D, E/E/E, and D/D/E in Vero-E6 cells. Time-course analysis shows that the isolated viruses exhibit different growth dynamics. The D/D/D strain exhibited the highest replication efficiency throughout the infection cycle, whereas the E/E/E strain showed the lowest replication levels. The D/D/E reassortant strain displayed an intermediate growth profile between the two parental genotypes. The superior replication capacity of D-type strains suggests that D-type genomic segments may confer enhanced viral adaptability and fitness across host environments. This phenotype–genotype correlation aligns with the predominance of genotype D observed in epidemiological surveillance, implying that its heightened replication efficiency may underlie its extensive geographic spread. It should be noted that each genotype was represented by a single isolate, and thus, the observed differences may reflect isolate-specific traits rather than generalizable genotype effects. Systematic studies involving multiple isolates per genotype are warranted to confirm these findings.

Comparative amino acid sequence analysis among D/D/D, D/D/E, and E/E/E strains further revealed that, relative to the E/E/E strain, the D/D/D strain harbored most of the amino acid residues previously associated with fatal clinical outcomes, as reported in previous study (29). Notably, reassortment involving the S segment from genotype E and the L and M segments from genotype D resulted in reduced replication efficiency, suggesting that the NP or NSs proteins encoded by the E-type S segment may exert a suppressive effect on viral replication. Detailed sequence comparison identified five amino acid substitutions (P207S, V223I, H245Q, H249Y, and L289P) in the NSs protein of the E-type strain relative to the D-type counterpart, four of which have been associated with fatal SFTS cases as well (29). These residues may play functional roles in modulating viral replication and pathogenicity, warranting further experimental validation. In addition, potential genotype-specific variations in RdRp and GP proteins should also be examined to better elucidate the molecular determinants underlying SFTSV replication diversity and virulence evolution.

It is noteworthy that recent SFTS outbreaks among fur-bearing animals in Shandong Province underscore their potential role in viral maintenance and cross-species transmission. In 2022, an outbreak of SFTSV infection occurred in a fur animal farm in Shandong, followed by subsequent epidemics in 2023 involving two farms in Weihai and six Arctic fox farms across Shandong and Liaoning Provinces (12, 15, 40). High homology of the viruses isolated from the patients, foxes, and environments provided genetic evidence for interspecies transmission. Notably, this was not an isolated incident. In January 2025, a human SFTS case in Weihai was linked to the consumption of fur animals that had died of disease. Virus samples isolated from this patient showed 98.7%, 99.1%, and 100% nucleotide sequence homology in the L, M, and S segments, respectively, with a local SFTSV strain transmitted by mink; these findings suggest a possible zoonotic link for SFTSV, particularly given the winter onset of the disease, the absence of familial clustering, and the clear history of exposure. These findings emphasize the need for integrated surveillance of fur animals to prevent zoonotic spillover and strengthen early warning for SFTSV transmission.

This study has several limitations. First, the genomic data set comprised only 24 sequences, which may not fully capture the genetic diversity of SFTSV circulating across Shandong Province. Second, replication kinetics were assessed solely in *vitro*, which may not accurately reflect viral pathogenicity or replication dynamics in natural hosts. Moreover, in the SFTS case linked to consumption of infected fur-bearing animals, neither tick samples nor animal-derived viral isolates were obtained. However, in June of the same year, an SFTSV strain highly homologous to the virus found in the patient was isolated from a mink farm located near the case; however, the nucleotide homology of its L fragment was only 98.67%. Furthermore, as no samples of the fur-bearing animals consumed by the patient were obtained, it has not yet been possible to directly confirm the source of transmission, and the relevant inferences remain subject to certain limitations. Given that only a single virus was purified for each genotype, the observed variation in viral growth kinetics may be attributable to individual strain characteristics rather than genotype-specific effects. Comprehensive evaluation of genotype-associated differences will require isolation of multiple strains per genotype and subsequent *in vivo* experimental validation. Despite these limitations, the study provides important insights into the genotypic diversity, reassortment, and host-associated evolution of SFTSV, offering a basis for understanding its zoonotic potential and informing future surveillance and functional studies.

In summary, this study provides comprehensive insights into the genetic diversity, evolutionary dynamics, and cross-species transmission potential of SFTSV in Shandong Province. Multi-species surveillance, whole-genome sequencing, and phenotypic analyses revealed the predominance of genotype D, which exhibited superior replication efficiency, likely contributing to its extensive geographic spread. Co-circulation of multiple genotypes facilitated genetic reassortment, particularly involving the S segment, while recombination events in L and M segments further enhanced viral diversity. Amino acid analyses highlighted mutations associated with pathogenicity and adaptation. Outbreaks in fur-bearing animals, coupled with highly homologous human and animal isolates, suggest a possible link to zoonotic diseases. Despite a limited genomic data set and *in vitro* replication assays, our findings underscore evolutionary plasticity and genotype-specific fitness of SFTSV, providing a foundation for integrated “human-animal-tick” surveillance, risk assessment, and targeted prevention strategies.

## Data Availability

Twenty-four complete SFTSV genome sequences from Shandong Province generated in this study have been deposited in GenBase at the National Genomics Data Center (NGDC). The accession numbers are as follows: C_AA120227.1–C_AA120250.1 for the S segment, C_AA120277.1–C_AA120300.1 for the M segment, and C_AA120301.1–C_AA120324.1 for the L segment. All sequences are publicly available at https://ngdc.cncb.ac.cn/genbase/. Detailed information on the SFTSV sequences used in this study is provided in Table 1.

## SUPPLEMENTAL MATERIAL

Supplemental legends spectrum.03573-25-s0001.docx Legends for Tables S1 to S3.

Supplemental data set spectrum.03573-25-s0002.xlsx Supplemental data set.

Table S1 spectrum.03573-25-s0003.xlsx Comparison of nucleotide and amino acid sequences for L, M, and S fragments of genotype D.

Table S2 spectrum.03573-25-s0004.xlsx Comparison of nucleotide and amino acid sequences for L, M, and S fragments of genotype E.

Table S3 spectrum.03573-25-s0005.xlsx Comparison of nucleotide and amino acid sequences for L, M, and S fragments of genotype F.

ASM does not own the copyrights to Supplemental Material that may be linked to, or accessed through, an article. The authors have granted ASM a non-exclusive, world-wide license to publish the Supplemental Material files. Please contact the corresponding author directly for reuse.
