# Supplementary material for: Phylogenetic and biological features of severe fever with thrombocytopenia syndrome virus from multi-species infections in eastern China
Source: Microbiol Spectr. 2026 Jul 13;14(8):e03573-25. doi: 10.1128/spectrum.03573-25 (PMC13436354; doi:10.1128/spectrum.03573-25)
Supplement: Supplementary file 1 [file spectrum.03573-25-s0001.docx]

**Supplementary information**

**Table S1. Comparison of nucleotide and amino acid sequences for L, M, and S fragments of genotype D.**

**Table S2. Comparison of nucleotide and amino acid sequences for L, M, and S fragments of genotype E.**

**Table S3. Comparison of nucleotide and amino acid sequences for L, M, and S fragments of genotype F.**
